# Understanding Knowledge and Attitude of Farmers towards Antibiotic Use and Antimicrobial Resistance in Jhunjhunu District, Rajasthan India

**DOI:** 10.3390/antibiotics12121718

**Published:** 2023-12-12

**Authors:** Virendra Singh Dhayal, Ayana Krishnan, Bilal Ur Rehman, Vijay Pal Singh

**Affiliations:** 1 Department of Biosciences, Shri Jagdishprasad Jhabarmal Tibrewala (JJT) University, Jhunjhunu 333001, India; vs.dhayal@nic.in; 2 CSIR-Institute of Genomics & Integrative Biology (CSIR-IGIB), Sukhdev Vihar, New Delhi 110025, India; ayana98pillai@gmail.com (A.K.); bilalurrehman13@gmail.com (B.U.R.); 3 Academy of Scientific and Innovative Research (AcSIR), Ghaziabad 201002, India

**Keywords:** antimicrobial resistance, veterinary antibiotics, antimicrobial stewardship program, one health, withdrawal period, antimicrobial use, antibiotic resistance, antibiotic sensitivity test

## Abstract

The misuse of antibiotics in veterinary practices by farmers is harming livestock production and food safety and leading to the rise of antibiotic resistance (AMR). This can also transfer resistant bacteria from animals to humans, posing a serious public health threat. However, we have not paid enough attention to understanding how farmers behave in this regard. Our study aims to explore farmers’ behaviors and identify the factors that influence their choices. To conduct this study, we used a questionnaire with 40 questions and surveyed 208 farmers in Jhunjhunu district, Rajasthan. We analyzed the data using SPSS. Here are the key findings: About 58.3% of the farmers have some awareness of antibiotics, and 49.5% are aware of antimicrobial resistance (AMR). Notably, as the level of education increases, so does awareness of antibiotics. Unfortunately, 63.9% of the farmers are not aware of the withdrawal time, and 64% have no idea about the presence of antibiotic residues during this period. Around 75% of farmers vaccinate their animals, but approximately 56.9% of individuals have never undergone an antibiotic sensitivity test (ABST) for milk. Around 48.6% of farmers are unaware of government testing centers. Several factors hinder farmers from implementing proper animal management practices, such as the high fees of veterinarians. When their animals become sick, their first choice is home remedies, followed by using old prescriptions. Additionally, 63.9% stop treatment once the animal looks better. A significant portion (83.8%) of farmers rely on local pharmacists for medicine. It has been determined that there is no significant correlation between education, experience, age, and the level of awareness concerning withdrawal periods, the existence of government antibiotic sensitivity test (ABST) centers, and entities responsible for sending samples for ABST. In our qualitative analysis, focus groups identified significant barriers to following best farm practices and spreading awareness about AMR. These findings suggest that addressing AMR in livestock requires a comprehensive approach. This should include targeted education and awareness programs for farmers, as well as improved access to veterinary services.

## 1. Introduction

Antimicrobial resistance (AMR) is the situation where pathogens/microorganisms develop resistance to an initially effective drug that no longer works against the same pathogen/microorganism. AMR has emerged as a critical global health concern that is on the rise, threatening the efficacy of medical treatments and the foundation of modern healthcare systems [1]. For more than 85 years, antibiotics have been used in animals, humans, and agriculture [2]. Along with its benefits, side effects, like AMR, led to prolonged illnesses, increased mortality rates, and higher healthcare costs [3]. The overuse and misuse of antibiotics have been identified as significant drivers of AMR. Irregular usage of antibiotics may make it more likely for microorganisms to evolve resistance. The workings of AMR need to be wholly understood and acknowledged. AMR cannot be stopped, but it may be controlled and made into a manageable issue [4]. The research in this field lacks appropriate data and models to estimate the resistant burden in food and animal production [5,6].

The workers who work close to livestock can be carriers for the environment and the food chain. Also, humans and animals excrete antibiotic residue that broadly spreads to local environments and other facilities and becomes the reason for resistant microorganisms [7]. Because of this, it is difficult and complex to find the link between use and misuse [8,9]. Antibiotics are vital for human and animal health. However, the misuse of antibiotics, poor surveillance of antibiotic use, and difficulties in achieving antimicrobial stewardship on the farm cause resistance in livestock [10]. Antibiotics are used in food-producing animals for various purposes, including therapeutic use, disease prevention, prophylaxis, meta-phylaxis, growth promotion, and infection treatment.

In the Indian context, where agriculture is a cornerstone of the economy and society, understanding the knowledge and attitudes towards antibiotic use and AMR is paramount. In 2010, the utilization of antimicrobial agents in food animal production amounted to approximately 63,151 tons. This figure is projected to surge by 67% by the year 2030, reaching a total of 105,596 tons [11]. Notably, two-thirds, or 66%, of this global upsurge in antimicrobial consumption can be attributed to the expanding population of animals being reared for food production [12]. By 2030, India is set to be among the top five nations with the highest proportion of global antimicrobial usage [12,13]. By 2030, India will be one of the five countries with the largest share of global antimicrobial consumption [14]. According to the 20th livestock census, the number of livestock is 535.8 million. Livestock also employs about 8.8% of the population in India [15]. The number of farmers is increasing worldwide, so knowing the entire population about AMR is essential. According to the OIE, 4 million human deaths were linked to AMR in 2019, of which 1.3 million were indirectly linked to AMR. Also, 60% of human pathogens originate from domestic or wildlife [16]. This depicts that animal, human, and environmental health are intrinsically intertwined and interdependent.

Antibiotic resistance has been linked to an extensive “over-the-counter” drug supply available to farmers and improper dispensing by para-veterinarians. Additionally, it increases access to antibiotics for those who may not have easy access to veterinarians. One way to control antibiotic abuse is to create awareness at the grassroots level, especially among low- and middle-income countries [17]. To address these circumstances, several initiatives have been created. In 2016, India launched a “Redline campaign” for antibiotic packaging. Common labeling standards will help the disciplined sale of antibiotics globally [18]. The Global Antibiotic Resistance Partnership (GARP) was formed to formulate practical policy proposals for nations with low and middle incomes [19]. Under the Drugs and Cosmetics Act (1940), sales and distribution of drugs in India are regulated, and antibiotic containers for the treatment of food-producing animals must have a withdrawal period mentioned for the species for which the drug is intended. However, it needs to be better followed in our country. The current veterinarian-to-cattle ratio is 1:4616 in India [20]. An insufficient number of veterinarians pushes farmers to depend on para-veterinarians. Studies reveal that they are 80% satisfied with para-vet services, with moderate to high satisfaction levels [21].

Rajasthan, where agricultural practices are influenced by traditional knowledge and modern techniques, directly impacts food security, livelihoods, and public health. Due to the predominantly agrarian economy within Rajasthan, using antibiotics in livestock farming is a common practice. It is integral to disease management; however, this practice concerns its potential contribution to AMR. While research on antibiotic use and AMR in healthcare settings has been extensive, there is a notable gap in understanding the perceptions and behaviors of farmers in agricultural contexts.

The AMR burden needs to be better studied in and in the livestock sector; there needs to be more evidence about the prescription practices, infection burden, and indications regarding the antibiotics prescribed in Rajasthan [22]. This research paper seeks to bridge this gap by investigating the knowledge and attitudes of farmers in the Jhunjhunu District towards antibiotic use and AMR. By examining the factors that influence antibiotic use practices, the level of awareness regarding AMR, and the underlying attitudes towards responsible antimicrobial stewardship, this study aims to provide a comprehensive understanding of the intricate interplay between agricultural practices and AMR.

### Rajasthan: Introduction

Rajasthan is the largest (one-tenth of the nation’s total land) Indian state located in the north-western part of the Indian subcontinent and comes second in overall livestock production (20th livestock population census). Around 10.60% of the total milk production in India is carried out in Rajasthan [23,24]. According to the last livestock census (20th conducted in 2019), the total population of cattle (including indigenous cattle and crossbreed cattle) and buffalo in Rajasthan is 3.85 lakhs and 5.21 lakhs, respectively.

## 2. Findings and Result

### 2.1. Socio-Demographic Details (Village, Age, Experience of Keeping Animals, Educational Qualification)

This study encompassed a total of 208 participants hailing from a diverse array of geographic locations and villages within the Jhunjhunu district. Notably, this represented a significant portion of the broader region, as our research covered 116 villages out of the total 927. All the individuals interviewed for this study were male farmers, constituting 100% of the surveyed population. Among the 208 participants, the age distribution was as follows: 34.1% (71/208) were below the age of 30 years, 24% (50/208) fell within the 31–40 year age bracket, 23.1% (48/208) were over 50 years old, and 18.8% (39/208) were aged between 41 and 50 years. The mean age of the participants was calculated to be 39.48 years (Table 1). In terms of educational attainment, 36.1% of the participants, which amounts to 75 out of 208, held a graduate degree. Additionally, 31.7% (66/208) had completed up to the 12th grade, while 6.7% (14/208) of the participants were found to be illiterate. For more comprehensive data, please refer to Table 1.

### 2.2. Number of Animals Involved, Type of Animal, and Work Experience

**Figure 1:**
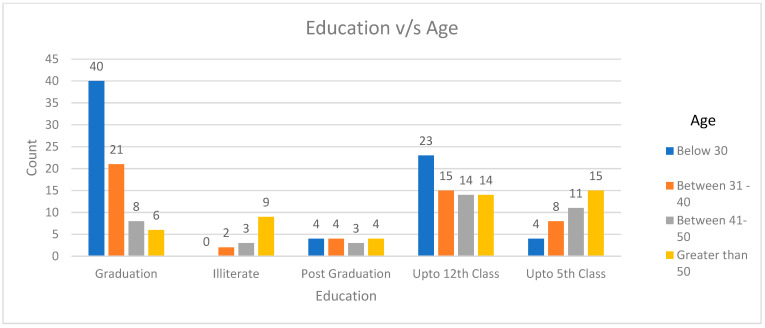
Comparing education to experience and age.

According to the data, a substantial 31% (166/208) of farmers are engaged in cattle farming, while 28% (154/208) are involved in buffalo husbandry, making livestock the dominant occupation among the participants. In terms of the number of animals they keep, nearly half of them, around 50.4% (105/203), have more than 5 animals in their care, with 27% (56/208) managing 6 to 10 animals and 16% (32/208) overseeing 11 to 20 animals (as detailed in Table 1). Notably, 20.6% (43/208) of the farmers qualify as big animal keepers, each tending to more than 10 animals. Their primary motivation for keeping animals is either for product sales, personal use, or a combination of both. Out of the total group of 208 farmers, approximately 33.7% have accumulated work experience since birth, another 33.6% have a work history spanning 11 to 30 years, and 23.1% have a work experience of 5 to 10 years, as indicated in Table 1. Out of a total of 208 participants, 75 individuals are graduates. Among these, approximately 53% (40/75) of the participants are under the age of 30, and approximately 41% (31/75) of them have had a native experience in their graduation (*p* < 0.001) (Figure 1).

### 2.3. The Level of Awareness Regarding Antibiotics and Antimicrobial Resistance (AMR) Is Correlated with One’s Educational Background, Work Experience, and Age

**Figure 2:**
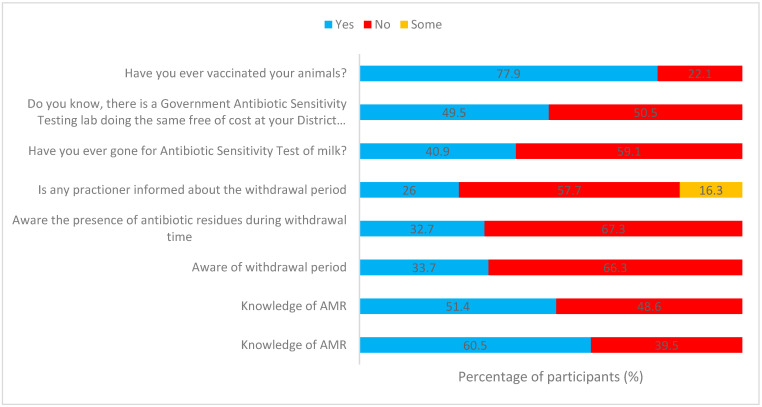
Distribution of the knowledge of farmers on antibiotics, antimicrobial resistance (AMR), withdrawal period, and antibiotic residues. (n = 208).

The distribution of farmers’ knowledge regarding antibiotics and antimicrobial resistance in the study regions is depicted in Figure 2. A substantial number of participants indicated that they were not familiar with what antibiotics are (82/208, 39.5%), and a significant portion (101/208, 48.5%) were unaware of antimicrobial resistance.

It is noteworthy that participants with higher levels of education were more likely to report knowing what antibiotics are and correctly understanding antimicrobial resistance, as outlined in Table 2. This difference in awareness was statistically significant with a p-value less than 0.001. Furthermore, these data indicated a direct correlation between increasing levels of education and heightened awareness regarding antibiotics and antimicrobial resistance. Out of the total sample, 75/208 (36%) graduates, 56/75 (74.7%), and 52/75 (69.3%) displayed knowledge about antibiotics and antimicrobial resistance (AMR). Conversely, among participants with limited education, such as those who were illiterate (14/208) or had completed only up to the 5th grade (38/208), totaling 52/208, only 18/52 (34.6%) were aware of antibiotics, and 12/52 (23%) were aware of AMR. These differences in awareness were found to be statistically significant (*p* < 0.001).

When assessing the impact of work experience on knowledge regarding antibiotics and antimicrobial resistance (AMR), it was observed that individuals with more extensive experience in cattle farming, both those who were native to the field and those with 11–30 years of experience, exhibited a stronger understanding of antibiotics and AMR. Specifically, out of 208 respondents, 70/208 (33.6%) had a native background, and 40/70 (57.1%) possessed knowledge about antibiotics. Among those with 11–30 years of experience, 35/208 (16.8%) had awareness of antibiotics, and 23/35 (65.7%) were familiar with them. These differences were found to be statistically significant (*p* < 0.001) (Table 2). However, these data did not reveal a substantial increase in awareness of AMR as work experience increased. Intriguingly, those with more extensive experience also exhibited a lower level of knowledge regarding AMR (*p* < 0.001), Table 2.

**Figure 3:**
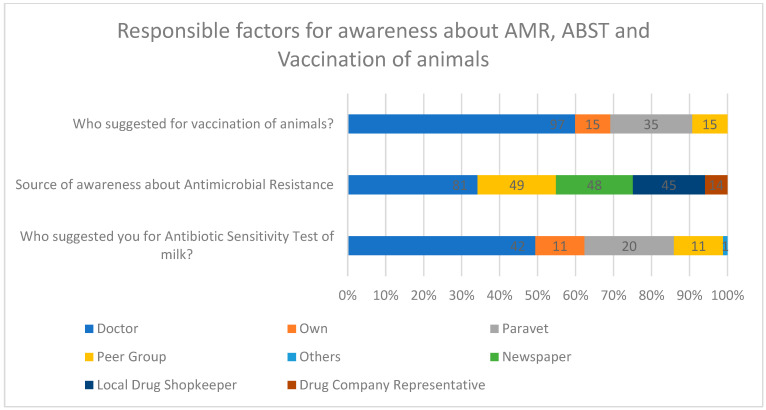
Responsible factors for awareness about AMR, ABST, and vaccination of animals.

In terms of age-related awareness, participants under the age of 30 displayed a higher level of knowledge regarding antibiotics. Out of the 208 participants, 71 (34.1%) are below the age of 30, and among them, 66.2% (47/71 individuals) possess knowledge about antibiotics (as shown in Table 2). Also for individuals aged between 41 and 50 (39/208), among them, 51.2% (20/39) had knowledge about antibiotics. It is noteworthy that age does not appear to correlate with awareness of AMR, as demonstrated by the nearly equal levels of awareness across all age groups, as indicated in Table 2. Awareness about AMR was primarily disseminated by doctors (46.6%), para-vet professionals (16.8%), and peer groups (7.21%) (as depicted in Figure 3).

### 2.4. Question: Have You Ever Gone for the Antibiotic Sensitivity Test of Milk?

**Figure 4:**
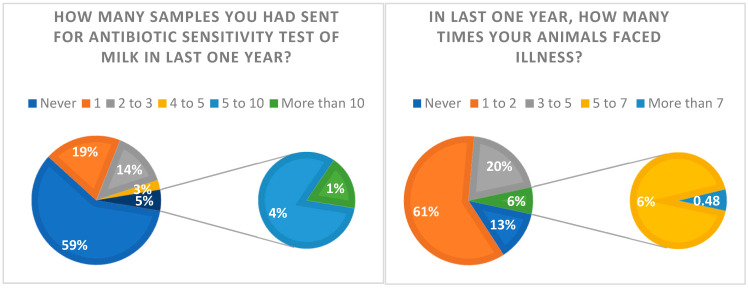
Comparison between frequency of animal became sick and number of samples sent for Antibiotic Sensitivity Test (ABST) of milk in last one year.

Out of 208 participants, 123 (59.1%) had their sick animals tested for antibiotic sensitivity (ABST), while 40.9% of farmers have never had ABST tests for their animals (Table 3). Additionally, 59% of farmers did not send any samples for ABST in the past year (Figure 4). More than half (61%) of the participants said their animals became sick 1 to 2 times in the past year. This means that 59% of farmers have never had ABST testing conducted, despite the high disease risk (Figure 4).

### 2.5. Question: Do You Know, There Is a Government Antibiotic Sensitivity Testing Lab Doing the Same Free of Cost at Your District Headquarters?

In response to the question Do you know that there is a government antibiotic sensitivity testing lab doing the same free of charge at your district headquarters? All participants are equally divided into two verticals, where almost half of them are aware (49.5%) of the government ABST laboratory, whereas the rest are unaware (50.5%) of the same (see Figure 2, Table 3). Regardless of their level of education, experience, or age, there is no significant improvement in awareness of government ABST testing centers (Table 3). This lack of improvement is consistent across various demographics. Doctors (49.5%), para-veterinarians (23.53%), and peer groups (12.94%) are the primary sources of information about ABST, highlighting the key influencers in disseminating knowledge (Figure 3).

### 2.6. Vaccination

Out of 208 participants, around 77.8% (162/208) chose to have their animals vaccinated, as opposed to 22.1% (46/208), *p* < 0.001, who did not (refer to Figure 2, Table 2 and Table 4). Surprisingly, a significant portion of the participants chose to undergo vaccination, and what is particularly noteworthy is that this majority includes individuals with lower levels of education and less experience (Table 3). Among those who did opt for animal vaccination, the motivation behind this decision varied. Specifically, veterinary doctors played a significant role, accounting for 59.88% of the motivations. Livestock assistants (para-veterinarians) were responsible for motivating 21.60% of farmers, while farmers’ peer groups influenced 9.26% of them. In 9.26% of cases, the farmers themselves took the initiative (as depicted in Figure 3).

Furthermore, in terms of the timing of their animals’ vaccination, 27.3% had their animals vaccinated six months ago, 16.2% three months ago, 15.7% a year ago, 13.0% nine months ago, and 2.8% more than a year ago (as detailed in Table 4).

### 2.7. Source of Medicine

In 181/208 (87%) of cases, the farmers have purchased medicines from the local pharmacist, whereas in 23/208 (11%) of cases, the farmers have used medicines that were left after the last treatment. Only in 1.92% of cases have farmers benefited from the government’s hospital or supply (Table 4).

### 2.8. Doctor’s Visit in the Last Year

As per the survey, 62.50% of farmers had called the doctor 1–2 times in the last year when the animal was sick. The doctor was called 3–5 times, 5–7 times, and more than 7 times in 20.19%, 5.77%, and 0.96% of cases, respectively. However, 10.58% of farmers do not even call the doctor for a single visit. This also indicates the higher disease burden among the animals, as in 88.46% of cases, the doctor was called multiple times (Table 4).

### 2.9. Distance of Veterinary Hospital from Your Residence (in km)

As per 132/208 (63.4%) of farmers, the facility of a veterinary hospital is available with a range of 1 to 3 km, whereas the distance of a veterinary hospital in 2.40% of cases is between 15 and 20 km. In 23.5%, 6.7%, and 3.8% of cases, the distance between the veterinary hospital and the farmer’s farm is 4 to 7 km, 7 to 10 km, and 10 to 15 km, respectively (see Table 4).

### 2.10. Checks Prior Treatment

There should be some checks and physical examinations by the practitioner before treating the animal. As per the responses of the current study, body temperature, ruminal movement, dry muzzle, pulse, and body weight were checked by the practitioner in 66.34%, 57.69%, 22.11%, 19.71%, and 15.86% cases, respectively. Further, in 73.55% of cases, either the animal was observed from a distance (21.63%) by the practitioner or treatment was provided based on symptoms indicated by the farmer (51.92%).

### 2.11. Question: Discontinuation of the Treatment or Prescribed Medicines/Antibiotic

As per the survey, once the animal recovered in appearance (139/208), 66.3% of farmers discontinued the treatment once the animal recovered in appearance and prescribed medicines, including antibiotics, whereas 29.8% discontinued the treatment only after consultation with the practitioner. Only 3.8% of the farmers continue the treatment until the complete consumption of medicines (Table 4).

### 2.12. Causes of Illness in Animals

A total of 20.19%, 6.25%, and 0.48% were informed that their animals fell ill 3 to 5 times, 5 to 7 times, and more than 7 times, respectively (Figure 4).

**Figure 5:**
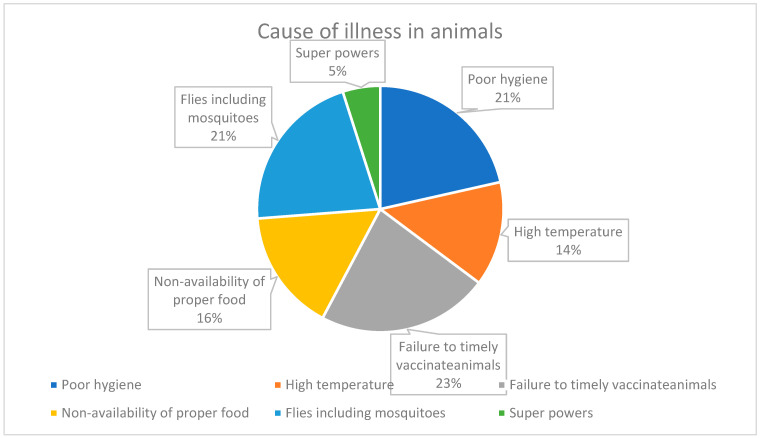
Causes of illness in animals.

23% of participants agreed that the major reason for the illness of animals is the failure to timely vaccinate them. Poor sanitation (21.48%), flies, including mosquitoes (21.30%), non-availability of proper food (16.02), and high temperatures (13.73) are among other reasons for animal illness (Figure 5). Surprisingly, as per 4.93% of participants, superpowers are the reason for animal illness.

### 2.13. Awareness about Antibiotics Used, Their Type, Withdrawal Period, and Presence of Antibiotics during the Withdrawal Period in Milk

As per the farmers, 54/208 (25.9%) of practitioners informed them about the withdrawal period, whereas 120/208 (57.7%) of them never talked about or informed them about the withdrawal period. In 34/208 (16.3%) of cases, some information was provided by the practitioners (Table 2). On almost the same trend, 30.29% of practitioners informed the farmer about the antibiotic used, its type, and the presence of the same in milk during the withdrawal period, whereas 56.25% did not (see Table 2). A total of 13.46% of practitioners inform the farmer about the treatment, up to some extent.

### 2.14. Question: Do You Know the Term “Withdrawal Period”? and Awareness of the Presence of Antibiotic Residues during Withdrawal Time?

Table 2 reveals that regardless of participants’ age, education, or work experience, there is a significant lack of knowledge regarding withdrawal periods and antibiotic residues. Out of a total of 208 participants, 138 (66.3%) individuals do not understand the term “withdrawal period”, and 140 participants (67.3%) are unaware of the existence of antibiotic residues during this period. When we look in detail, among graduates, 47/75 (62.6%) did not know the term “withdrawal period”, and 46/75 (61.3%) were unaware of the presence of antibiotic residues during this period. Post-graduates did not fare much better, with 12 out of 15 (80%) unaware of withdrawal time and 11 out of 15 (73.3%), *p* < 0.001, not knowing about antibiotic residues during withdrawal time. Even among illiterates, 11 out of 14 (78.6%) did not understand the term “withdrawal time” and the presence of antibiotic residues during that period (Table 2). Surprisingly, regardless of experience, none of the participants were aware of withdrawal time and antibiotic residues during withdrawal time. Among native farmers, 55 out of 70 (78.6%) did not know of withdrawal time, and 53 out of 70 (75.7%) were unaware of antibiotic residues during withdrawal time. The trend continued across different experience levels, with 27 out of 48 (56.3%) of those with 5–10 years of experience and 14 out of 20 (70%), *p* < 0.001, with less than 5 years of experience not knowing these concepts. The same pattern was observed with age (Table 2).

In summary, only 33.7% of farmers had an understanding of the “Withdrawal Period”, and 32.7% were conscious of the presence of antibiotics in milk during this period, while the majority, 66.3% and 67.3%, respectively, were not aware (Table 2). These data suggest that factors like age, education, and experience are not reliable indicators of awareness regarding withdrawal time and antibiotic residues. Interestingly, doctors played a significant role in raising awareness about antimicrobial resistance, vaccination, and antibiotic sensitivity tests for milk, as shown in Figure 3 and Table 2.

### 2.15. Question: “Do You Feel Sometimes Your Animals Are Not Recovered, Even after a Lot of Efforts by Veterinarians/Para-Veterinarians?”

In response to the question, 67.79% of farmers either agreed or somewhat agreed with it. On the other hand, 25.00% rarely agreed, while 7.21% disagreed with the statement (see Table 3). The data in Table 3 suggests that as education levels go up and age goes down, farmers tend to have more trust in veterinarians and para-veterinarians.

**Figure 6:**
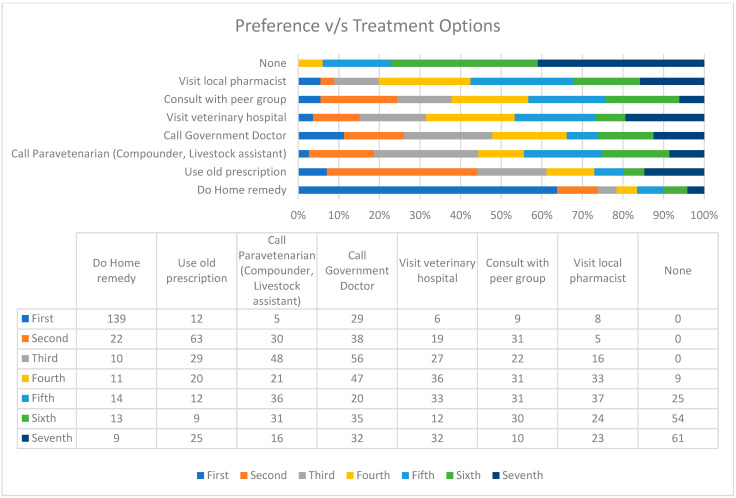
Farmer’s priority during animal illness.

When an animal is unwell, around (139/208), 66.8% of farmers prefer using home remedies as their first priority, while only (63/208), 30.2%, consider using old prescriptions as their second choice. Additionally, (56/208), 26.9% of farmers opt for calling a government doctor as their third preference (Table 4, Figure 6). Also, farmers complained that the main reasons for these preferences are the high fees charged by doctors, trust in home remedies, and the desire to reduce animal healthcare costs. This may involve using homemade remedies, like decoctions, to treat animal illnesses over an extended period of time. The ingredients of decoctions include carrom seeds (*ajwain)*, akshan, dry ginger (*Saunth*), heart-leaved moonseed (*giloy*), dashmool (a combination of the roots of 10 ayurvedic herbs), etc. The other home remedies include serving cereals (wheat, pearl millet (*baajra*), fenugreek (*methi*), etc.), oil, jaggery, steaming with sugar, and carrom seeds (*ajwain)*. The home remedy also includes some superstitious methods, such as steaming with a mixture of an old woman’s hair and mustard oil.

## 3. Materials and Method

### 3.1. Study Area and Population (Figure 7)

**Figure 7:**
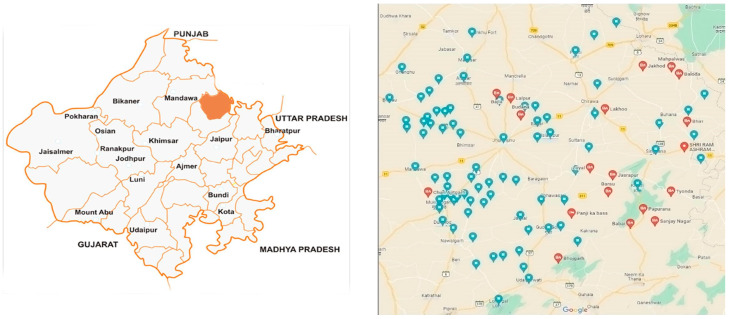
Rajasthan District Map.

The target respondents of the survey were cattle farmers who had at least one cattle and were residents of the Jhunjhunu district of Rajasthan, India (Figure 7). The current study was conducted between November 2021 and June 2022 to investigate the awareness of antibiotics and AMR among cattle farmers, the preference of cattle farmers during animal illness, and the cattle farmers’ views and consciousness about the prescription practices of veterinarians/livestock assistants, practicing in the study area. The current study was implemented as an online survey among cattle farmers residing in the Jhunjhunu district. Since no study of this type has been conducted yet, the study area as well as the area were chosen.

### 3.2. Interview Design, Piloting, and Sampling

We administered a survey using traditional pen-and-paper methods to collect participant feedback in the local language (Hindi), which was subsequently transcribed into a Google Form and Excel for more accessible validation of the responses. This involved soliciting written notes of the responses provided by the participants and gathering responses from 208 cattle farmers across 116 villages in the Jhunjhunu district. The survey comprised three sections with a total of 40 questions, including general, closed-ended, open-ended, and multiple-choice questions. The first section focused on socio-demographic details, encompassing information such as name, village, age, experience in animal husbandry, educational qualifications, and related aspects. The second section delved into awareness-related inquiries, exploring the participants’ knowledge about different types of animals, their awareness regarding antibiotics and antimicrobial resistance (AMR), and the sources of this information. Finally, the last section comprised behavioral questions. It investigated reasons attributed to animal illnesses, priorities when seeking treatment for animals, and the prescription practices followed by veterinarians or livestock assistants in the region.

### 3.3. Statistical Data Analysis

The survey data included binary response options for knowledge and practice items, while the attitude questions were structured as open-ended questions with varying answers. We initially stored the collected data in Microsoft Excel 2019 and subsequently conducted an in-depth analysis using IBM SPSS (IBM Statistics for Windows Version 29.0.1.0 (171)). To gain insights into the relationships between knowledge, attitude, and practice concerning antibiotics and antibiotic resistance (AMR), as well as their associations with various independent variables, we employed the Chi-square test. More specifically, the Chi-square test was utilized to uncover factors that were associated with a strong comprehension of antibiotic resistance and usage, as well as attitudes toward these critical topics. We established a two-sided *p*-value threshold of 0.001 to determine statistical significance. In the final model, covariates with *p*-values less than 0.001 were included, while those with *p*-values greater than 0.001 were excluded. Ultimately, we organized and presented the analyzed data in tables, graphical representations, and a narrative format that effectively communicated the findings.

### 3.4. Ethical Consideration

A formal ethics approval (Ref No: CSIR-IGIB/IHEC/2021-22/02 Dt. 08.02.2022) was obtained from the Institutional Human Ethics Committee of the CSIR-Institute of Genomics and Integrative Biology in New Delhi for the conduct of this study. This study was conducted with the consent of all participants.

## 4. Discussion

The global impact of AMR on animal and human health is a matter of concern [25]. Veterinarians and farmers are the crucial factors that promote and limit the development of AMR [26]—only very few studies have assessed a farmer’s perspective on AMR in India. Antibiotics are essential for animal health and increase their life span [27]. To enhance the effectiveness of antibiotics, India’s public health authorities must implement intervention strategies that consider the risk perception of antimicrobial resistance (AMR). This qualitative study aims to enhance comprehension of the factors impacting antibiotic usage among farmers in livestock production. This sheds light on the interconnected nature of misunderstandings and the various factors associated with the use and effectiveness of antibiotics. These results stress the importance of dealing with these problems to effectively tackle the increasing issue of antibiotic resistance in farms caused by a lack of awareness and misunderstandings.

Our study successfully involved individuals of different age groups, including younger and older farmers, ensuring diverse perspectives and experiences. These farmers keep various types of animals, reflecting the region’s agricultural diversity. Cattle (31%) and buffalo (28%) are the most common animals they rear. However, our findings reveal that many of these farmers lack a good understanding of antibiotics (38.0%) and antimicrobial resistance (AMR) (46.8%) (see Figure 2), despite more than 90% of them being literate. This suggests that literacy sometimes equates to a better understanding of AMR [28]. However, basic education can help them comprehend how antibiotics function and their impact on populations. However, studies indicate that people with at least a high school education tend to understand antibiotics better [29].

The survey results indicate a correlation between demographic figures such as age, education, and experience in respondents’ understanding of antibiotics and AMR, aligning with Ozturk’s 2019 findings [29]. Moreover, it’s evident that mere awareness of antibiotics doesn’t guarantee responsible usage. Some respondents knew about antibiotics but lacked awareness of AMR, underscoring the necessity to enhance health literacy. Such literacy plays a pivotal role in fostering a comprehensive understanding of antibiotics, AMR, and their proper utilization.

Given that there’s no substitute for antibiotics, employing them judiciously becomes imperative. Critical use involves administering the correct dosage at the appropriate time, adhering to withdrawal periods, and following prescribed guidelines [30]. This approach ensures their efficacy and longevity. The uniformity in perspectives expressed by nearly all participants echoes the consistency found in Ozturk’s study, emphasizing the importance of continual efforts to bolster health literacy and responsible antibiotic use within communities.

Further findings include the fact that the treatment based on ABST is relatively low. Around 56.9% of farmers have never gone to ABST. Nearly half of the participants need to be made aware of the government ABST lab doing it for free (Table 2). So, distance and unawareness about government facilities can be reasons for the low ABST count. A total of 34.14% responded that the average distance between the veterinary hospital and the farmer’s farm is 4–15 km. It reveals that there is no easy access to hospitals when in need. Taking animals to the hospital will add an extra expense, too. Hence, there is a need to draw some attention to the lack of infrastructure. The Ministry of Animal Husbandry and Dairying has introduced mobile veterinary units (MVUs), intending to deliver veterinary services directly to farmer’s doorsteps [31,32]. Government officials will ensure such services reach the grass-roots level and have to be systematically monitored. Our research shows that veterinarians and para-vets are crucial in spreading awareness about vaccination, antibiotic stewardship (ABST), and antimicrobial resistance (AMR). Animal health authorities should also be actively involved in informing people about these topics and enhancing the availability of facilities while promoting responsible antibiotic use (ASP) [33]. According to the National Action Plan India, ICMR implemented ASCIP programs in 2012; AMSP will help in improving diagnostic facilities and implementing infection control programs, thus decreasing the number of situations where the urgency of antimicrobial prescription is high [34].

Our findings reveal that there should be improvement in good animal management practices in the livestock sector. In the survey, participants agreed that most diseases are caused by not vaccinating (23%), poor sanitation, and mosquitoes (42.78%) (Figure 5). Also, surprisingly, a tiny percentage stated some superpowers involved. Animals are more susceptible to disease since they live in stressful environments [35]. Farmers and animals live in close contact in developing countries like India, along with the infectious disease burden [36]. Poor sanitation can cause cross-contamination, and the workspace itself becomes a ground for breeding resistance [37]. Giving more importance to disease prevention by ensuring good hygiene will eventually decrease the need for antibiotics. The farmers could not provide proper hygiene, which may be due to a lack of disinfectant and proper shelter. Most farmers own more than 20 animals, and most opt for poor hygiene, making their animals sick. If they do not maintain hygiene in such crowds, animals’ health is at stake, hence the need for more antibiotics. Along with implementing awareness regarding the importance of sanitation, funding should also be provided. For example, the Ministry of Fisheries, Animal Husbandry, and Dairy initiated Assistance to States for Control of Animal Diseases (ASCAD) to ensure surveillance and related activities, including funding to farmers for losses due to infection [38].

In India, the Department of Animal Husbandry and Dairying implemented a livestock health and disease control scheme to improve animal health through prophylactic vaccination. From our findings, nearly 75% of people are aware of vaccinations, which is a good indicator. Still, 21.3% (46/209) of them neglected or never vaccinated their animals. Immunization could benefit health and mortality. Secondarily, it will limit the inappropriate use of antibiotics each year. Also, it is an excellent alternative to antibiotics [39]. For example, the vaccination against *Lawsonia intracellularis*, in Danish pigs can reduce oxytetracycline consumption for the condition by 80% [39]. However, the vaccine is expensive for rural farmers and may not be easy to access for them. According to the findings, in 88.46% of cases, the doctor was called multiple times. This indicates a higher disease burden among the animals. These results can directly correlate with poor hygiene, animals being sick very often, and the percentage not opting for vaccination.

Further, considering our studies, most participants purchased medicines from the local medical storekeepers. This indicated that they believe antibiotics are a cost-effective solution to protect animal health and maximize their life expectancy and profit. Farmers may find it simple to rely on their local pharmacy for antibiotics because they likely do not require a prescription. Another reason to depend on local shops must be the distance between the veterinary hospital and the farmers’ place. Private antibiotic access should be restricted through strict prescription audits to help curb poor antibiotic prescribing [40]. Notably, in countries like India and Bangladesh, a substantial 95% of medications are dispensed by local pharmacies. However, this statistic may significantly vary in rural settings like Jhunjhunu [41].

Worldwide studies have shown that farmers trust professional advice from veterinary experts [42,43]. However, this advice often comes at a cost. Veterinary professionals earn money through various means, such as prescribing medications, charging for their administration, consultation fees, follow-up treatments, and covering production expenses. This aligns with the findings of our study, where farmers expressed faith in doctors but were hesitant to seek their help when their animals became sick due to high fees. Additionally, our results indicate that farmers rarely doubt the efforts of veterinarians and para-vets, with only 16.2% feeling that these efforts are insufficient. Therefore, veterinarians must enhance their services by effectively communicating with farmers and fostering trust. This aligns with findings from Svensson (2022) [44], which also underscore the importance of such actions.

When the animal is not well, the priority in around 64% of cases is the farmers’ home remedy (kaddha). As per the responses, the major reasons to indicate the above-stated preferences by farmers include the doctor’s higher fee, faith in the doctor, minimizing the maintenance cost of the animal, and the higher cost of animal treatment. Another problem with the home remedy is that kaddha has shown short-term side effects in humans, like heartburn, constipation, and a decreased RBC count [45]. In India, home remedies/herbal medicine (kadhai) are considered traditional and indigenous medicine and made with the ingredients available at home [46]; hence, they are cost-effective, easily dosable, and believed to be safe. However, they are unsure about the side effects, effectiveness, and safety of animals. For 30.7%, calling a government doctor or visiting a hospital is the last priority, probably due to the abovementioned reasons. By this time, the animal’s condition can also go out of control or may not respond to medications anymore.

Some farmers (11.06%) have used leftover medicines from previous treatments, and only 1.6% go to government hospitals for medicines. While 61.1% have access to a veterinary hospital within 1–3 km, it is surprising that many still prefer to buy medicine from local pharmacists. This suggests that the government supply of veterinary medicine may not meet the demand, leading to higher animal maintenance costs. The scarcity of government-supplied medicines also results in over-the-counter sales and direct marketing of veterinary antibiotics to farmers. Local pharmacists often lack professional training and are eager to sell medicines without requiring a prescription. This poses a problem, as they may not fully understand how to handle these medicines [47]. Some farmers may not be well-informed about antibiotics and fall into the category of those who rely on local pharmacists, potentially obtaining antibiotics without realizing the risks of antibiotic resistance. Moreover, the fact that 10.6% of farmers continue to use leftover medicines from prior treatments highlights a concerning practice that can contribute to developing antibiotic resistance (AMR) in cattle. This improper use of antibiotics without proper guidance or supervision raises significant AMR risks within the livestock sector [48].

The response to checking animals before starting treatment by the veterinarian shows empirical therapy. Most practitioners solely rely on body temperature, observed from a distance; treatment is provided based on symptoms indicated by the farmers. All these practices reflect the misuse, overuse, and inappropriate use of antibiotics. The body temperature of cattle can increase or decrease throughout the day, so no one can claim that an animal is sick or not by checking its body temperature [49]. The cow’s surroundings have a significant impact on its body temperature. The time of day and the cow’s activity also affected the cow’s core temperature [50]. So, the temperature cannot determine whether the animal is sick. According to OIE norms, there should be some checks and physical examinations by the practitioner before treating the animal. Observing the animal from a distance or providing treatment based on symptoms described by the farmer is an irrational practice and a potential chance for AMR.

This study identified an important issue that farmers poorly respect and observe: 66–67% are unaware of the withdrawal period and the fact that antibiotic residue may be in milk during the withdrawal period. Similarly, 66.65% of farmers discontinue the prescribed antibiotics once the animal recovers in appearance. This can cause a drive to AMR. Almost the same findings were reported by Mutua in 2020 [51,52]. Few studies have been conducted in India on milk and milk products with antibiotic levels exceeding the limit [53,54]. A small percentage of this study also reveals the sale of milk under antibiotic treatment. The main reason for highlighting the presence of antibiotics in milk is due to not observing withdrawal time. A study in Australia reveals that successful observation of the withdrawal period in goat milk has considerably decreased the oxytetracycline count in milk [55]. This study also reveals that farmers complain that around 56–57.69% of practitioners never talked about the withdrawal period, an antibiotic used, its type, or its presence in milk. Awareness and understanding of AMR are the first strategic objectives [56,57], So, informing our farmers of withdrawal times and promoting ASP/Infection control programs to fellow veterinarians is essential. Awareness explaining the importance of “responsible use” will provide information about the rational use of antibiotics among farmers who are not professionally educated. Also, it can curb the inappropriate use of antibiotics by building awareness among consumers and providers (veterinary professionals, para-vets, and local shopkeepers) [58,59]. A study in Kerala has found that state-wise education, training, and coordination between various civil communities (e.g., Accredited Social Health Activists (ASHA)) through antimicrobial stewardship programs significantly increased public health and showed success in Nipah and novel coronavirus outbreaks [60,61]. The survey results reveal a spectrum of viewpoints within the farming community regarding the effectiveness of veterinary interventions. While many express concerns about animals’ full recovery, others hold more optimistic views. This variation offers a rich ground for future research and collaboration between farmers and veterinary experts, aiming to enhance animal health and well-being.

Our questionnaire survey was determined to investigate cattle farmers’ awareness of antibiotics and AMR. An antimicrobial stewardship program should be provided for farmers to eradicate all the gaps in this study. This will improve diagnostic facilities and awareness and help organize infection control programs and carefully coordinate through various channels, including schools, accurate information disseminated by the media, public awareness campaigns, and interactions between veterinarians and farmers [51]. Enforcement of laws and legislation will help restrict the sale of antibiotics. In this way, farmers will depend more on government dispensaries than local medical shops. Hence, there must be systematic monitoring through prescription audits and sales of antibiotics to help minimize overuse and misuse of antibiotics.

## 5. Conclusions

This study offers valuable insights into farmers’ behaviors and knowledge gaps, shedding light on the multifaceted factors contributing to antimicrobial resistance (AMR). Most farmers exhibit a limited understanding of antibiotics and AMR, with even those aware of the issue often needing more up-to-date information. While there is moderate awareness, significant knowledge gaps persist, with higher education levels correlating with greater awareness. Most farmers are unaware of essential aspects of antibiotic use, such as withdrawal times and the presence of antibiotic residues during the withdrawal period. This underscores the pressing need for comprehensive training on proper antibiotic application. Although vaccine utilization is relatively high, antibiotic sensitivity testing remains less common. Educating farmers about the benefits of sensitivity testing is beneficial.

Additionally, many farmers lack awareness of government testing centers and report that veterinarians do not adequately advise on withdrawal periods. Addressing these communication gaps through enhanced outreach and guidance from veterinarians is crucial. Farmers prioritize home remedies and old prescriptions, highlighting the necessity for clear treatment protocols. The prevalent dependence on local pharmacies for medication indicates a need for improved access to veterinary care and oversight. Economic constraints, including high veterinary fees, also impact farmers’ behavior. Supporting farmers with grants and resources can facilitate the adoption of better animal management practices and, subsequently, reduce antibiotic use.

In conclusion, this study underscores the need for multifaceted interventions to combat AMR in the livestock sector. Targeted education, improved veterinary services, economic incentives, and policy measures should collectively promote responsible antibiotic use. Farmers’ knowledge gaps and behavior drivers revealed here can be the foundation for effective interventions. A comprehensive approach is required, involving education and awareness campaigns for farmers and collaboration among government bodies, veterinarians, retailers, and other stakeholders to implement effective antimicrobial stewardship practices. This holistic strategy is essential for preserving the cattle industry’s sustainability and safeguarding one’s health, ensuring the well-being of both animals and humans.

## 6. Limitations of the Study

The information gathered only relates to one Indian district. Studying a single area might not provide a clear picture of the country’s AMR situation. The results of this study cannot be generalized, but they do provide a glimpse into the gaps and scenarios that exist at the grassroots level.

## Figures and Tables

**Table 1:** Socio-demographic details of participants.

Parameter	Value
Age, years, n ^$^ (%)	
Below 30	71 (34.1)
Between 31 and 40	50 (24)
Between 41 and 50	39 (18.8)
Greater than 50	48 (23.1)
Mean age, years	39.48
Work Experience, n (%)	
By Birth	70 (33.7)
Less than 5	20 (9.6)
Between 5 and 10	48 (23.1)
Between 11 and 20	35 (16.8)
Between 21 and 30	35 (16.8)
Highest Qualification, n (%)	
Illiterate	14 (6.7)
Up to 5th Class	38 (18.3)
Up to 12th Class	66 (31.7)
Graduation	75 (36.1)
Post-graduation	15 (7.2)
Keeping (type of animal), n (%)	
Buffalo	154 (28)
Cattle (Cow)	166 (31)
Goat	122 (22)
Sheep	34 (6)
Dog	61 (11)
Poultry	1 (0.18)
Number of animals, n (%)	
1 to 2	44 (21)
3 to 5	59 (28)
6 to 10	56 (27)
11 to 20	32 (16)
21 to 30	11 (5)
More than 30	6 (3)
Use of animal products, n (%)	
Own use	70 (33.7)
For sell	21 (10.1)
Both	115 (55.3)
Other purposes	2 (1.0)

^$^ Number of participants.

**Table 2:** Showing the association of age, educational status, and work experience with knowledge of antibiotics and knowledge of AMR, aware of the withdrawal period, aware of the presence of antibiotic residues during withdrawal time, the practitioner informed them about the withdrawal period or not.

Status		Do You Know What Is an Antibiotic?			Are You Aware of Antimicrobial Resistance?			Do You Know the Term “Withdrawal Period”			Aware of the Presence of Antibiotic Residues during Withdrawal Time			Is Any Practitioner Informed about the Withdrawal Period			
Level of Education	n (%)	Yes (n/126)	No (n/82)	*p*-Value	Yes (n/107)	No (n/101)	*p*-Value	Yes (n/70)	No (n/138)	*p*-Value	Yes (n/68)	No (n/140)	*p*-Value	Yes (n/54)	No (n/120)	Some (n/34)	*p*-Value
Illiterate	14 (6.7%)	4 (3.2)	10 (12.2)	<0.001	2 (1.9)	12 (11.9)	<0.001	3 (4.3)	11 (8.0)	<0.001	3 (4.5)	11 (7.9)	<0.001	3 (5.5)	11 (9.2)	0	<0.001
Up to 5th class	38 (18.4%)	14 (11.1)	24 (29.3)		10 (9.3)	28 (27.7)		7 (10.0)	31 (22.5)		7 (10.4)	31 (22.7)		6 (11.1)	25 (20.8)	7 (20.6)	
Up to 12th class	66 (31.7%)	41 (32.5)	25 (30.5)		34 (31.8)	32 (31.7)		29 (41.4)	37 (26.8)		25 (37.3)	41 (29.3)		23 (42.6)	31 (25.8)	12 (35.3)	
Graduation	75 (36.0%)	56 (44.4)	19 (23.2)		52 (48.6)	23 (22.8)		28 (40)	47 (34.1)		29 (42.6)	46 (32.9)		11 (32.4)	44 (36.7)	20 (37.0)	
Post Graduation	15 (7.2%)	11 (8.7)	4 (4.9)		9 (8.4)	6 (5.9)		3 (4.3)	12 (8.7)		4 (6.0)	11 (7.9)		2 (3.7)	9 (7.5)	4 (11.8)	
**Total**	n = 208	126 (60.5)	82 (39.5)		107 (51.4)	101 (48.6)		70 (33.7)	138 (66.3)		68 (32.7)	140 (67.3)		54 (26)	120 (57.7)	34 (16.3)	
**Work Experience**																	
Between 11 and 20	35 (16.2)	23 (18.3)	12 (14.6)	<0.001	21 (19.6)	14 (13.9)	<0.001	16 (22.9)	19 (13.8)	<0.001	15 (22.4)	20 (14.3)	<0.001	12 (22.2)	20 (16.7)	3 (8.8)	<0.001
Between 21 and 30	35 (16.2)	19 (15.1)	16 (45.7)		15 (14.0)	20 (19.8)		12 (17.1)	23 (16.7)		12 (17.9)	23 (16.4)		11 (20.4)	20 (16.7)	4 (11.8)	
Between 5 and 10	48 (22.2)	30 (23.8)	18 (22.0)		27 (25.2)	21 (20.8)		21 (30.0)	27 (19.6)		19 (28.4)	29 (20.7)		16 (29.6)	25 (20.8)	7 (20.6)	
Less than 5	20 (9.3)	14 (11.1)	6 (7.3)		11 (10.3)	9 (8.9)		6 (8.6)	14 (10.1)		5 (7.5)	15 (10.7)		3 (5.6)	10 (8.3)	7 (20.6)	
Native	70 (32.4)	40 (31.7)	30 (36.6)		33 (30.8)	37 (36.6)		15 (21.4)	55 (39.9)		17 (24.0)	53 (37.9)		12 (22.2)	45 (37.5)	13 (38.2)	
**Total**	n = 208	126 (60.5)	82 (39.5)		107 (51.4)	101 (48.6)		70 (33.7)	138 (66.3)		68 (32.7)	140 (67.3)		54 (26)	120 (57.7)	34 (16.3)	
**Age**																	
Below 30	71 (32.9)	47 (37.3)	24 (29.3)	<0.001	37 (34.6)	34 (33.7)	<0.001	21 (30.0)	50 (36.2)	<0.001	22 (31)	49 (35.0)	<0.001	16 (29.6)	35 (29.2)	20 (58.8)	<0.001
Between 31 and 40	50 (23.1)	32 (25.4)	18 (22.0)		31 (29.0)	19 (18.8)		25 (35.7)	25 (18.1)		24 (35.8)	26 (18.6)		19 (35.2)	26 (21.7)	5 (14.7)	
Between 41 and 50	39 (18.7)	20 (15.9)	19 (23.2)		16 (15.0)	23 (22.8)		10 (14.3)	29 (21.0)		9 (13.4)	30 (21.4)		7 (13.0)	28 (23.3)	4 (11.8)	
Greater than 50	48 (22.2)	27 (21.4)	21 (25.6)		23 (21.5)	25 (24.8)		14 (20.0)	34 (24.6)		13 (19.4)	35 (25.0)		12 (22.2)	31 (25.8)	5 (14.7)	
**Total**	n = 208	126 (60.5)	82 (39.5)		107 (51.4)	101 (48.6)		70 (33.7)	138 (66.3)		68 (32.7)	140 (67.3)		54 (25.0)	120 (55.6)	34 (15.7)	

**Table 3:** Showing the association of age, educational status, and work experience with the vaccination status of animals, Have you ever gone for an antibiotic sensitivity test on milk? Do you know that there is a government antibiotic sensitivity testing lab doing the same free of charge at your district headquarters? Do you feel that sometimes your animals are not recovered, even after a lot of efforts by veterinarians/para-veterinarians?

Status		Have You Ever Vaccinated Your Animals?			Have You Ever Gone for an Antibiotic Sensitivity Test of Milk?			Do You Know, That There Is a Government Antibiotic Sensitivity Testing Lab Doing the Same Free of Cost at Your District Headquarters?			Do You Feel That Sometimes Your Animals Are Not Recovered, Even after a Lot of Efforts by Veterinarians/Para-Veterinarians?				
**Level of Education**	n (%)	Yes (n/162)	No (n/46)	*p*-value	Yes (n/85)	No (n/123)	*p*-value	Yes (n/103)	No (n/105)	*p*-value	Yes (n/35)	No (n/15)	Sometimes (n/106)	Rarely (n/52)	*p*-value
Illiterate	14 (6.5%)	12 (7.4)	2 (4.3)	<0.001	4 (4.7)	10 (8.1)	<0.001	3 (2.9)	11 (10.5)	<0.001	0 (0.0)	1 (6.7)	11 (10.4)	2 (3.8)	<0.001
Up to 5th class	38 (17.6%)	26 (16.0)	12 (26.1)		15 (17.6)	23 (18.7)		16 (15.5)	22 (21.0)		5 (14.3)	0 (0.0)	25 (23.6)	8 (15.4)	
Up to 12th class	66 (30.6%)	56 (34.6)	10 (21.7)		30 (35.3)	36 (29.3)		35 (34.0)	31 (29.5)		14 (26.9)	4 (26.7)	34 (32.1)	14 (40.0)	
Graduation	75 (34.7%)	56 (34.6)	19 (41.3)		31 (36.5)	44 (35.8)		44 (42.7)	31 (29.5)		14 (40.0)	7 (46.7)	30 (28.3)	24 (46.2)	
Post Graduation	15 (6.9%)	12 (7.4)	3 (6.5)		5 (5.9)	10 (8.1)		5 (4.9)	10 (9.5)		2 (5.7)	3 (20.0)	6 (5.7)	4 (7.7)	
**Total**	N = 208	162 (77.9)	46 (22.1)		85 (40.9)	123 (59.1)		103(49.5)	105 (50.5)		35 (16.8)	15 (7.2)	106 (51)	52 (25)	
**Work Experience**															
Between 11 and 20	35 (16.2)	26 (16.0)	9 (25.7)	<0.001	18 (21.2)	17 (13.8)	<0.001	19 (18.4)	16 (15.2)	<0.001	4 (11.4)	1 (6.7)	20 (18.9)	10 (19.2)	<0.001
Between 21 and 30	35 (16.2)	28 (17.3)	7 (20.0)		17 (20.0)	18 (14.6)		17 (16.5)	18 (17.1)		2 (13.3)	2 (5.7)	22 (20.8)	9 (17.3)	
Between 5 and 10	48 (22.2)	37 (22.8)	11 (23.9)		21 (24.7)	27 (22.0)		27 (26.2)	21 (20.0)		6 (17.1)	4 (26.7)	22 (20.8)	16 (30.8)	
Less than 5	20 (9.3)	15 (9.3)	5 (10.9)		8 (9.4)	12 (9.8)		11 (10.7)	9 (8.6)		3 (20.0)	3 (8.6)	9 (8.5)	5 (9.6)	
Native	70 (32.4)	56 (34.6)	14 (30.4)		21 (24.7)	49 (39.8)		29 (28.2)	41 (39.0)		20 (57.1)	5 (33.3)	33 (31.1)	12 (23.1)	
**Total**	N = 208	162 (77.9)	46 (22.1)		85 (40.9)	123 (59.1)		103 (49.5)	105 (50.5)		35 (16.8)	15 (7.2)	106 (51)	52 (25)	
**Age**															
Below 30	71 (32.9)	54 (33.3)	17 (37.0)	<0.001	24 (28.2)	47 (38.2)	<0.001	36 (35.0)	35 (33.3)	<0.001	13 (37.1)	7 (46.7)	25 (23.6)	26 (50.0)	<0.001
Between 31 and 40	50 (23.1)	39 (24.1)	11 (23.9)		26 (30.6)	24 (19.5)		31 (30.1)	19 (18.1)		7 (20.0)	2 (13.3)	28 (26.4)	13 (25.0)	
Between 41 and 50	39 (18.1)	29 (17.9)	10 (21.7)		13 (15.3)	26 (21.1)		13 (12.6)	26 (24.8)		7 (20.0)	1 (6.7)	24 (22.6)	7 (13.5)	
Greater than 50	48 (22.2)	40 (24.7)	8 (17.4)		22 (25.9)	26 (21.1)		23 (22.3)	25 (23.8)		8 (22.9)	5 (33.3)	29 (27.4)	6 (11.5)	
**Total**	N = 208	162 (77.9)	46 (22.1)		85 (40.9)	123 (59.1)		103 (49.5)	105 (50.5)		35 (16.8)	15 (7.2)	106 (51)	52 (25)	

**Table 4:** Responses to the various statements.

Variables	n (%)
Have you ever vaccinated your animals?	
Yes	162(77.8)
No	46(22.1)
Time since the last vaccination was performed	
12 months	34 (15.7)
3 months	35 (16.2)
6 months	59 (27.3)
9 months	28 (13.0)
More than 12 months	6 (2.8)
Source of medicine	
Government hospital	4 (1.9)
Local Pharmacist	181 (87)
Remaining out of the last treatment	23 (11)
Distance of the veterinary hospital from your residence	
1–3	132 (63.4)
4–7	49 (23.5)
7–10	14 (6.7)
10–15	8 (3.8)
15–20	5 (2.4)
When your animal is ill, what will you do?	
Home Remedy	139 (66.8)
Use an old prescription.	63 (30.2)
Call a government doctor.	56 (26.9)
Generally, when you discontinue the prescribed medicines/antibiotics	
Once the animal recovered in appearance	138 (66.3)
After consultation with a practitioner	62 (29.8)
Complete consumption of medicines	8 (3.8)
In the last one year, how many times have you called a doctor?	
1–2 times	130 (62.5)
3–5 times	42 (20.2)
5–7 times	12 (5.8)
More than 7	2 (0.96)
None	22 (10.6)

## Data Availability

The raw data of this study will not be readily available to ensure the confidentiality of the participating cattle farmers as it may contain potentially identifiable information. The data that supported the authors decision to write this article will be made available by the authors as and when required.
